# The role of auditory feedback in the motor learning of music in experienced and novice performers

**DOI:** 10.1038/s41598-022-24262-x

**Published:** 2022-11-17

**Authors:** Maria Giovanna Luciani, Alessandra Cortelazzo, Alice Mado Proverbio

**Affiliations:** 1 Cognitive Electrophysiology Laboratory, Department of Psychology, University of Milano-Bicocca, Piazza Dell’Ateneo Nuovo 1, 20126 Milan, Italy; 2 Francesco Venezze’ Music Conservatory of Rovigo, Rovigo, Italy

**Keywords:** Neuroscience, Psychology

## Abstract

Musical learning is related to the development of audio-visuomotor associations linking gestures with musical sounds. To study the role of auditory feedback in learning, 115 students (56 guitarists, 59 pianists) at the beginner, intermediate and advanced levels were recruited. Playing with sound (audio-motor feedback), mute practice (motor feedback), and piece listening (auditory feedback) were compared to first sight reading to assess the role of auditory and motor feedback in procedural learning. The procedure consisted of the execution of a standard piece for determining the students’ level and 4 further music executions (every week for 4 weeks), preceded by different practice conditions (for 12 min, once a day, for 5 days). Real musical pieces (e.g., Segovia, Schubert, Bartók) were used. Performance evaluation focused on four macro-categories: note, rhythm, dynamics and smoothness. For both instruments, first-sight reading (A − M −) was associated with the worst performance: silent motor practice (A − M +) resulted in learning the rhythmic structure of the piece and in a smoother performance. Listening to pieces (A + M −) resulted in learning the agogics and in improving articulation and smoothness. Listening during performance (A + M +) resulted in fewer intonation errors. Interestingly, auditory feedback was more relevant for beginners than for advanced students, as evidenced by the greater benefits of listening during practice.

## Introduction

When playing a musical instrument, auditory feedback is strongly involved in movement control processes, leading to a close coupling between perception and action^1,2^. Experienced musicians possess complex and highly sophisticated multimodal skills, including the joint processing of auditory, proprioceptive and visuomotor data, to be used in the regulation of extremely fine movements^3,4^. For this reason, there exist closely associated auditory and motor neural circuits in the musician’s brain. Furthermore, musical learning involves the formation of new audio-visual-motor associations^5,6^. Indeed, transcranial magnetic stimulation (TMS)^7^, electroencephalogram (EEG)^8^, magnetoencephalography (MEG)^9^ and functional magnetic resonance (fMRI)^2^ studies have shown that listening to a music piece that one knows how to play activates brain regions involved in its motor representation. Conversely, watching hands playing on a keyboard in the absence of sound stimulates the auditory and premotor cortices (e.g. ^6,10^). These findings indicate that music training promotes the emergence of cross-modal associations between action and perception^11–13^. The study of a musical instrument leads to cortical reorganization in response to the increasing need for multisensory processing, and in particular to the development of an audio/motor network^14^.

According to Novembre^15^, during musical performance, internal operational models developed by musicians during the years of training would be able to predict auditory outcomes of ongoing motor commands by comparing them with afferent copies (auditory feedback). Therefore, during the execution of a musical sequence, images of the ‘predicted’ sounds (formed long before the actual production of the sound) are compared to the actual result in real time. Therefore, self-monitoring during musical performance is constant and necessarily relies upon rapid feedforward and feedback control systems that link the auditory target with the movements necessary for its production^16^. Once the auditory-motor representation is established, the predicted or imagined sounds of the learned melody activate motor representations. In feedforward interactions, the auditory system would influence motor output in a predictive manner, thus allowing the motor planning of predictable actions, supporting motor preparation, facilitating error detection and correction, and guiding perception. Such mechanisms mainly involve the temporo/parietal audio-motor network^7^. Feedback mechanisms are particularly relevant for string instruments, wind instruments and singing, where intonation needs to be constantly monitored and corrected if it is out of tune^17,18^.

Some studies have specifically investigated the contribution of acoustic feedback during learning a piece of music. Herroio Ruiz et al.^19^ examined self-monitoring systems in musicians by testing the electrophysiological correlates of executive control mechanisms during error production. A group of pianists was tested while executing previously learned musical pieces at high speed in the presence or absence of auditory feedback. They aimed to study the interplay between auditory and sensorimotor information triggered by errors during performance. The data showed that, independent of auditory feedback, approximately 70 ms before an error was committed, a negative response generated within the anterior cingulate cortex was observed, possibly reflecting the prediction of an incoming error and correction processes. According to Herroio Ruiz and co-authors^19^, during musical execution, the actual outflow of the movement would be compared with the provided motor command so that in the event of a mismatch, an error signal would be triggered to remedy the incorrect movement. This model of auditory feedback is compatible with the findings by Finney and Palmer^20^, who reported how the absence of auditory feedback did not worsen the performance of memorized musical sequences in musicians. Again, the model agrees with the view expressed by Pfordresher and Palmer^13^, highlighting the need for experienced pianists to largely anticipate the preparation of rapid movements through motor planning. To further explore the role of auditory feedback in music learning, Repp^21^ carried out a study in which a group of pianists was asked to play a musical fragment under four conditions: expressive with and without feedback and in metronome time with and without feedback. The data showed that pianists were capable of generating the expressive timing pattern of their performance in the absence of auditory and kinesthetic feedback. Apparently, expressiveness was generated from an internal representation of the music that did not depend on auditory feedback, but it was much reduced or absent when kinesthetic feedback from the piano keyboard was eliminated. Again, Brown and Penhune^22^ investigated the role of auditory feedback in pianists engaged in the learning of a simple melody (followed by its recall) under 4 learning conditions: listening (auditory learning), performing without auditory feedback (motor learning), performing with auditory feedback (auditory-motor learning), or observing the musical score without performing or listening. Surprisingly, participants showed greater accuracy in piece execution (in terms of notes and rhythm) in auditory learning than in motor learning. It was hypothesized that musicians might use powerful feedforward motor control systems developed through practice and used during auditory learning to transform sensory information into motor commands. However, it should be mentioned that the stimuli for this study (12 melodies) were not musical pieces but a very elementary combination of five unique pitches (12 notes in all) that could be played using the right hand in a fixed position with one finger per piano key (a sort of finger tapping). Therefore, it cannot be excluded that melodies’ extreme motor similarity led auditory information to be essential for their learning (more than motor signature).

While much valuable knowledge is available on the role of auditory feedback in the learning of simple melodies, little is known about the role of auditory and motor feedback in the learning of real and challenging music pieces. Furthermore, it is not known whether their reciprocal contribution changes as a function of students’ music proficiency. Indeed, previous investigations have shown that the internal representation of musical sounds and related motor gestures become progressively more robust and refined after many years of experience^23,24^. For example, a difference was found between groups of pianists with 3000 versus 10,000 h of piano study behind them^25^ in the magnitude of error-related response triggered by the observation of incongruent audio-motor gestures. The ability to detect an incongruence between a musical gesture and its related/unrelated sound was found to increase linearly with the number of years of academic study^26^. Consequently, one should expect acoustic feedback to play a very relevant role during the first three to five years of musical study, when these multimodal connections have not yet been formed.

The present study aimed to shed light on some aspects not fully investigated by previous literature. The performance of pianists and guitarists was compared after one week of studying novel pieces in different conditions: through auditory learning, mute motor learning, audio-motor learning, and no learning at all (first-sight reading). Students belonged to six different age and class ranges, from novice to experienced performers. Instead of short basic fragments, here real, 16 bars long, classical music pieces, unfamiliar to students, were used as stimuli to ensure the authenticity of learning processes. The randomization of pieces and learning conditions across students helped eliminate the risk of learning across conditions (unlike in Repp’s^21^ study, in which the same excerpt was used in the different conditions), as well as the risk that the technical peculiarities of the pieces would influence the amount and type of errors made during their execution. The general goal of the study was to assess the role of auditory and motor feedback in learning how to play new pieces. The auditory feedback was manipulated by muting the instruments or by listening to recorded performances of the pieces, while the motor practice was manipulated by allowing or impeding actual playing on the instruments.

Overall, acoustic feedback (A + M +) was expected to improve motor learning^22,27^ compared to silent motor practice (A − M +). Furthermore, it was hypothesized that repeatedly listening to a piece (A + M −) could contribute to implicit learning compared to the first sight condition (A − M −). Again, pure motor practice (A + M −) was expected to improve rhythmic performance because brain areas involved in motor processing (e.g., cerebellum and basal ganglia) are also responsible for controlling the tempo and rhythm of actions^28^. According to our hypotheses, audio-motor practice (A + M +) would result in fewer intonation errors than pure motor practice because repeated audio-motor practice is likely to reinforce the audio-motor representation of musical sounds, thus improving the ability to play in tune^23^. Based on the findings of Herroio et al.^19^, auditory feedback was expected during motor learning to be more relevant for inexperienced learners than for advanced learners. Again, based on Repp^21^, it was expected that learning without auditory feedback (silent motor learning) would not completely impede the learning of expressiveness (tempo, agogics, articulation, etc.).

## Materials and methods

### Participants

The participants were 115 music students (59 pianists and 56 guitarists; 63 males and 52 females; see Table 1 for details) aged between 11 and 32 years (mean age = 19.6, SD = 6.4). The sample size was superior to that recommended by G*Power 3 analysis^29^ with an α = 0.01. Most participants were recruited from music conservatories. All provided written informed consent; informed consent was also obtained from the parents of the minor participants. All participants and parents also signed a release form to allow the video recording of the musical performances. Informed consent was obtained from the parents for the publication of identifiable images in an online open-access journal. The project was approved by the Ethics Committee of University of Milano-Bicocca (protocol number RM-2021-370) and by the directors of the Conservatories or Civic Music Schools. All methods were performed in accordance with the relevant guidelines and regulations. The participants were divided into three subgroups (beginners, intermediate and advanced) according to their level of proficiency, the course they attended at the Conservatoire, the age at which they began their musical studies, and the difficulties of the repertoire they were used to studying. Proficiency was the key factor, also assessed through the execution of a level pièce.

**Table 1:** Demographic and academic characteristics of participants. F = female, M = male. YAS = years of academic musical studies. G = Guitarists; P = Pianists. The six subgroups were merged into three level categories, Beginners, Intermediate and Advanced, for the purpose of statistical analysis. The mean age and YAS are reported, along with standard deviation values and minimum and maximum values.

Level	N	Sex	Age	YAS	Instrument
Beginners I	18	7 F, 11 M	11.7 (SD = 1.85) (9–15)	2.6 (SD = 1.09) (1–5)	9G, 9P
Beginners II	23	7 F, 16 M	14.8 (SD = 3.22) (9–23)	4.7 (SD = 1.96) (2–9)	9 G, 14 P
Intermediate I	20	10 F, 10 M	19.1 (SD = 3.84) (12–29)	8.6 (SD = 2.76) (4–13)	11 G, 9 P
Intermediate II	19	6 F, 13 M	24.1 (SD = 5.55) (17–35)	12.2 (SD = 4.63) (3–23)	10 G, 9 P
Advanced I	18	5 F, 13 M	24.4 (SD = 4.55) (18–34)	15.1 (SD = 5.86) (8–26)	9 G, 9 P
Advanced II	17	7 F, 10 M	25.5 (SD = 2.83) (22–30)	16.1 (SD = 3.88) (11–24)	8 G, 9 P
Group/mean	115	42 F, 73 M	19.7 (SD = 6.39) (9–35)	9.7 (SD = 6.14) (1–26)	56 G, 59 P

All students were right-handed as assessed through the Edinburgh Handedness Inventory. They had normal or corrected-to-normal vision and normal hearing, as self-reported. All participants reported no history of learning or reading deficits or neurological, psychiatric or motor disorders. To test the experimental procedure and setting, four additional pilot subjects, two guitar students and two piano students, were preliminarily tested.

### Stimuli

**Figure 1:**
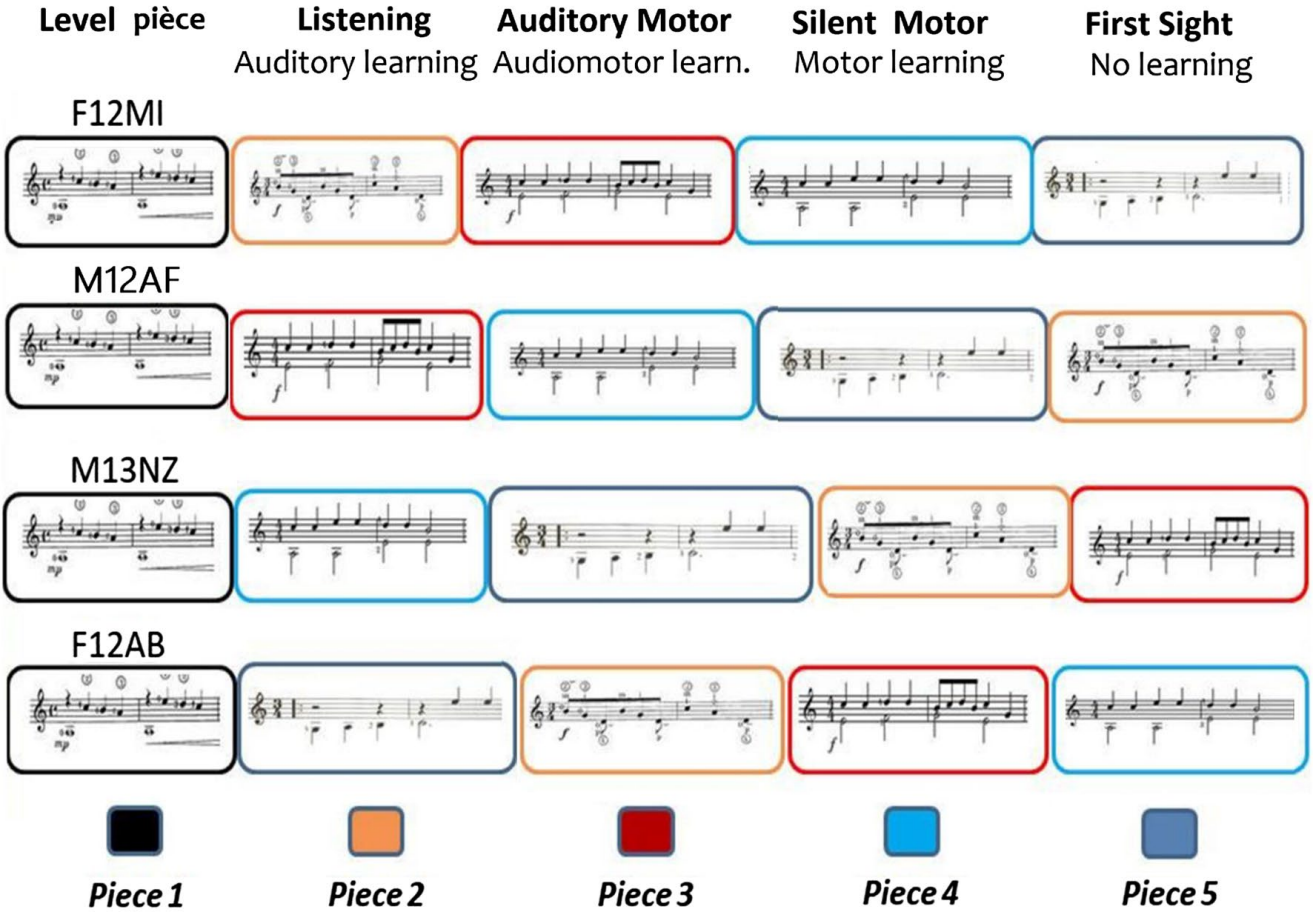
Example of piece randomization across the four experimental conditions for four students belonging to the same didactic level (beginners). The effectiveness of musical learning was evaluated in the same students under different practice conditions (on different pieces) and among students with different levels of proficiency. The same musical pieces (here indicated by the same colour, red, blue, etc.) were assigned to different study conditions across participants of the same level.

This investigation comprised four experimental conditions, preceded by a session in which the student was evaluated while playing a defined ‘level’ piece, which was the same for all students at the same level of expertise, to ascertain their correct grade. All students completed all four conditions in random order, while the execution of the level piece always occurred on the first meeting. Students were randomly assigned to a specific order of learning sessions and received instruction on how to study the musical pieces assigned from week to week. Learning was based on “listening” to the assigned piece (A +) without looking at the score, motor practice on the instrument while listening to the music (“audio-motor”, A + M +) and reading the score, silent motor practice with the muted instrument (“motor-silent”, M +) while reading the score, or no study at all (“first-sight”, A − M −). For all students and levels of expertise, selections of real studies of exactly 16 bars in length were selected (some examples are visible in Appendix 1). Pieces were of equivalent technical difficulty across instruments and levels of expertise. Pieces of the same level were selected within similar study collections and music volumes. The piece difficulty increased as the student’s expertise increased. Independent evaluators, including Conservatory teachers, assessed the difficulty equivalence between the proposed pieces within each sublevel of proficiency: guitar pieces were validated by four musicians with guitar degrees, while piano pieces were validated by four musicians with piano degrees. Pieces lasted between 37″ and 1 min 30″ and were chosen from piano or guitar study collections (e.g., by Segovia, Shubert, Bartok music books, etc.). Pieces were randomized (among students of the same level) across the four learning conditions (see Fig. 1).

### Procedure

The experimental sessions were scheduled one week apart to allow the students to study in the meantime and were video-recorded. A written script was used for interacting with students and instructing them. The first session was devoted to the administration of the Oldfield inventory and to the recording of the level piece execution. The next four sessions involved the execution of the previously assigned pieces. After the performance, for which students were always praised and encouraged, regardless of the performance, instructions were given about the study modality for the new piece in one of the four experimental conditions. Its execution took place the following week. The procedure was as follows:

#### Level piece

The students were asked to sit ready to play on the instrument (which had been previously tuned) in front of the camera, in a position where both hands could be clearly seen. They first took a quick look at the score (while the experimenter timed them for one minute) and started playing at first sight, after an initial moment of concentration. The instructions were to concentrate on the music without worrying about possible mistakes and to continue playing (without starting over or repeating the passage) in case of mistakes. All students were encouraged to reduce performance anxiety.

#### Listening condition

Students were instructed to listen (through headphones) to a recorded performance of the assigned piece (provided by the experimenter), repeatedly, for exactly twelve minutes, once a day, for five days, with the recommendation of not reading the music sheet. The following week, they were asked to perform the piece in the same modalities described above.

#### Audio-motor condition

Students were instructed to study the assigned piece for twelve minutes, once a day, for five days, as they used to do (i.e., playing and listening to the music). The following week, they were asked to perform the piece.

#### Motor silent condition

Students were instructed to study the assigned piece for twelve minutes, once a day, for five days, with a mute instrument. For guitars, a soft cloth was inserted under the strings, halfway between the sound hole and the bridge, to block the string vibrations. Digital pianos were easily silenced for this type of learning. The following week, they were asked to perform the piece.

#### First sight

No study of a piece was required for this condition. On the day of the performance, students were asked to execute a piece at first sight, after a quick look, at moderate speed, not worrying much about possible mistakes and not interrupting the performance until the end.

**Figure 2:**
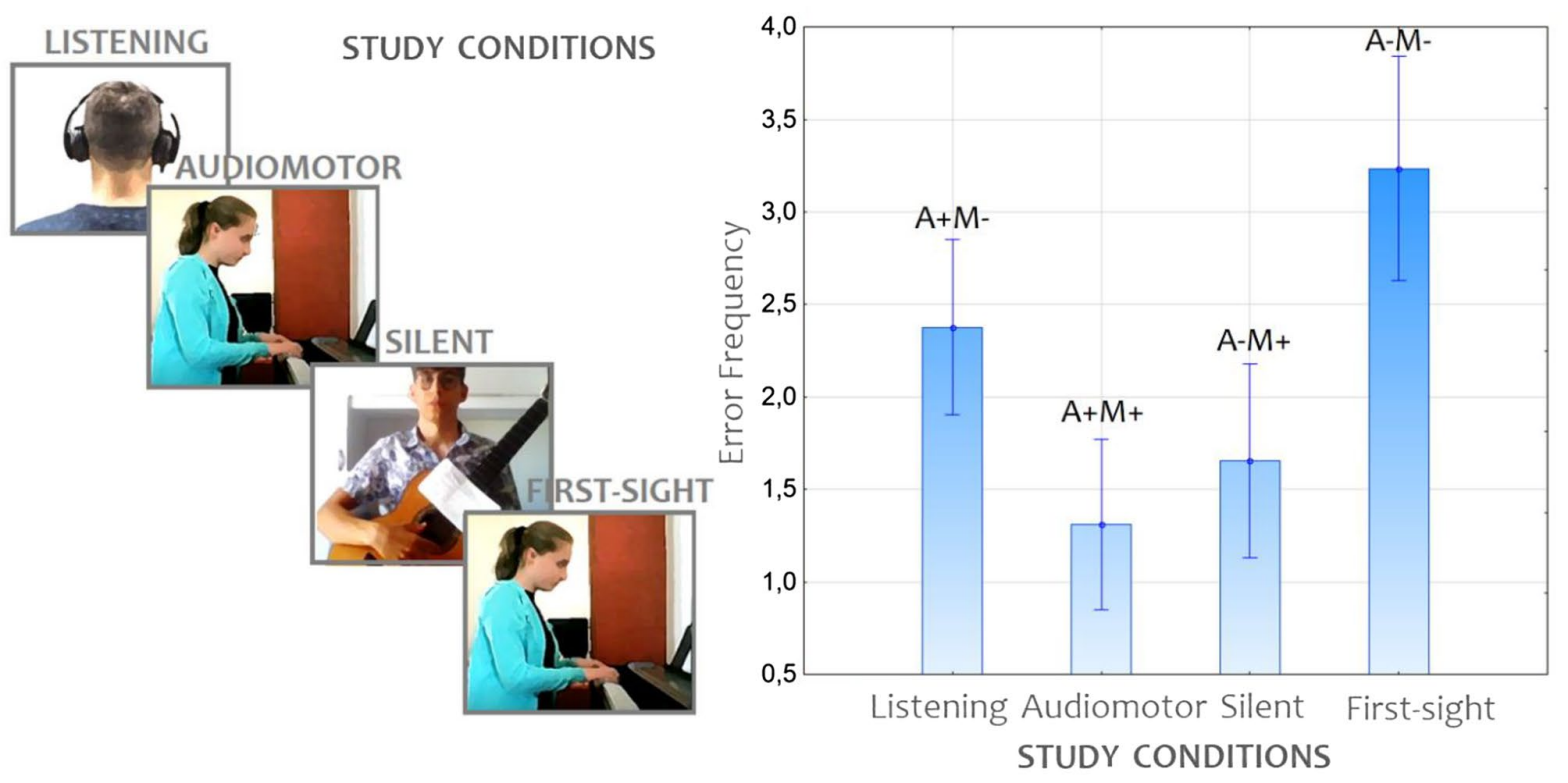
(Left) Example of session order, which was rotated across participants. (Right) A description of the experimental variables modulated across the different learning conditions is provided and applied to the real data for simplicity. Mean error frequency was observed in the four experimental conditions as a function of the sensory modality involved in music learning. M +  = motor practice, M −  = lack of motor practice, A +  = auditory feedback, A −  = lack of auditory feedback. Error bars indicate standard errors.

The order of learning conditions was randomized across students, with the exception of the execution of the level piece that always took place in the 1st session (see Fig. 2 (left) for an example).

The performances of the students were independently and anonymously evaluated in a double-blind manner by four teachers (two females and two males, aged between 23 and 44 years) with extensive teaching experience and/or artistic expertise in the two instruments. Evaluators were unaware of the learning conditions linked to a specific performance. Videos were saved with an alphanumerical code, and only after the whole scoring process were they linked to the specific experimental condition.

The technical parameters applied as evaluation criteria covered four major types of error:

- Note error (playing a wrong note, i.e., a wrong or out-of-tune pitch). Error type = N
- Errors in the reproduction of rhythmic figures. Type of error = R
- Failure to respect dynamics (e.g., piano, mezzo-forte, sforzando), tempo (agogics) and signs of articulation (staccato, legato, pizzicato, puntato, cadenza, rallentando, ritornello, etc.). Type of error = D
- Lack of smoothness, fluidity and regularity of performance (stumbling, interruptions, repetitions). Type of error = S

The number of errors associated with a given execution by a single student was represented by the mean of the errors reported by the teachers. When a given note/musical gesture concurrently contained two or even three types of errors (for example pitch, rhythm, and dynamics), the evaluator pinned all three types of errors on the evaluation grid.

The performance of the students during the execution of the level piece was not analysed based on the four dimensions, as it had not been preceded by a specific practice but served to confirm the level of the students, established in advance by their teachers, and to familiarize them with the procedure and the experimenters.

In the listening condition, participants used an in-ear headset with a jack. 3.5 mm SONY model Mdr-Ex15Ap e JBL Model T110. Piano student performed on these instruments (all had 88 weighted keys, 3 pedals: damper, sostenuto, soft): Yamaha C3 half-tail piano or Yamaha U3 vertical piano, Yamaha CLP-725, Casio AP-Celvian. During the silent motor condition, students playing the electronic piano shut it down during practice. Those who used the traditional piano were required to put a blanket between the strings and the hammers.

The guitarists used 6 classic strings, 4/4 size guitars of this type: Alhambra 3C, Salvador Cortez CC-10, Yamaha C70. During the silent motor condition, all the guitarists blocked the strings by placing four masks under the strings at the 12°fret.

### Data treatment and statistical analysis

The number of errors committed by each student in the four experimental conditions was subjected to a repeated-measures ANOVA, whose variability factors were instrument (piano, guitar), level (beginner, intermediate, advanced), learning condition (listening, audio-motor, silent-motor, first sight), and error type (note, rhythm, dynamics, fluency). Tukey post hoc comparisons among means were carried out. Statistical analyses were performed using *Statistica* (Stat Soft TIBCO) software.

## Results

**Figure 3:**
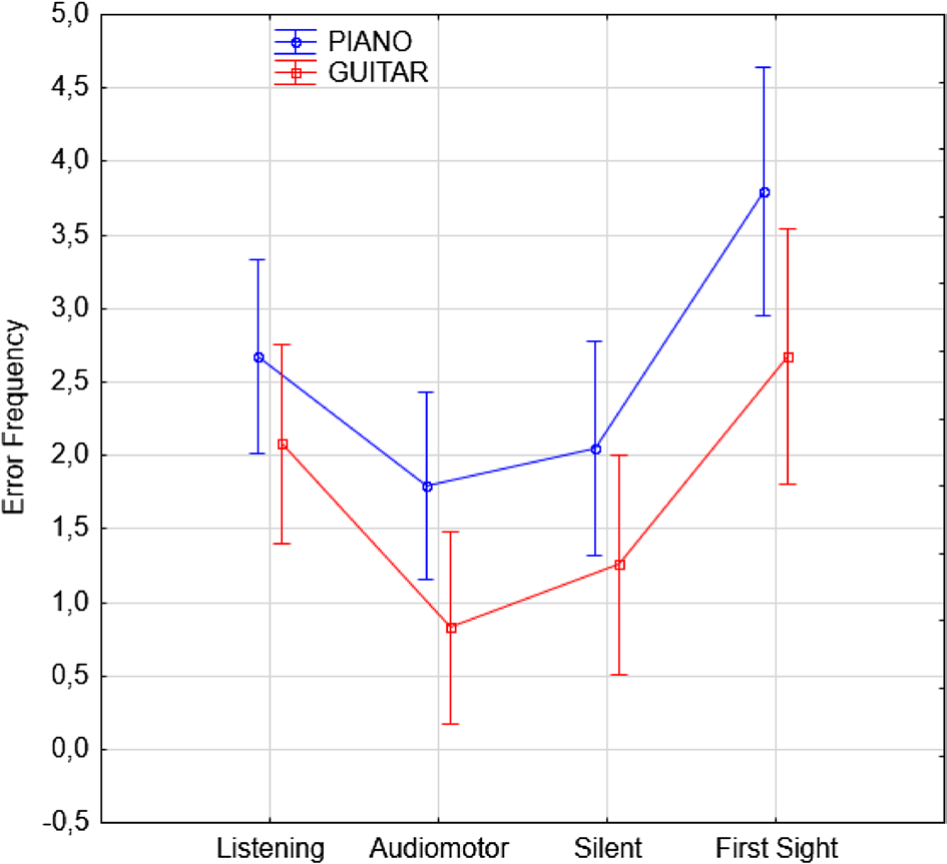
Error frequency observed for the two musical instruments and as a function of the learning condition. Error bars indicate standard errors.

The ANOVA showed the significance of the instrument factor [F(1, 109) = 20.11; *p* < 0.001], with pianists committing overall more errors (2. 58, SE = 0. 14) that guitarists (1. 71, SE = 0. 14), as shown in Fig. 3.

The level factor was also significant [F(1, 109) = 4.32; *p* < 0.015]. Post hoc comparisons (*p* = 0.037) showed that beginners made overall more errors (2.51, SE = 0.16) than advanced students (2.10, SE = 0.17), while intermediate students made the fewest errors (1.83, = 0.17; *p* < 0.001). The ANOVA showed the further significance of the study condition [F(3, 327) = 70.08; *p* < 0.001], with the highest error rate observed in the first-sight condition (3.23, SE = 0.31) and the lowest in the audio-motor condition (1.31, SE = 0.23), as can be observed in Fig. 2 (right) and in Fig. 3. Overall (independent of error type, students’ level and instrument played), the error rates observed both in the listening (2.38, SE = 0.24) and silent-motor conditions (1.65, SE = 0.26) were significantly lower than those observed in the first-sight condition (*p* < 0.001) but higher than those observed in the audio-motor condition. In turn, fewer errors (*p* < 0.001) were committed in the silent-motor than in the listening condition (*p* < 0.001).

The error type factor also yielded significance [F(3, 327) = 9.71; *p* < 0.001]. Smoothness errors were the most frequent (2.67, SE = 0.33), followed by dynamics errors (2.22, SE = 0.26) and note errors (2.16, SE = 0.41), with the least frequent error involving rhythm (1.52, SE = 0.28).

**Figure 4:**
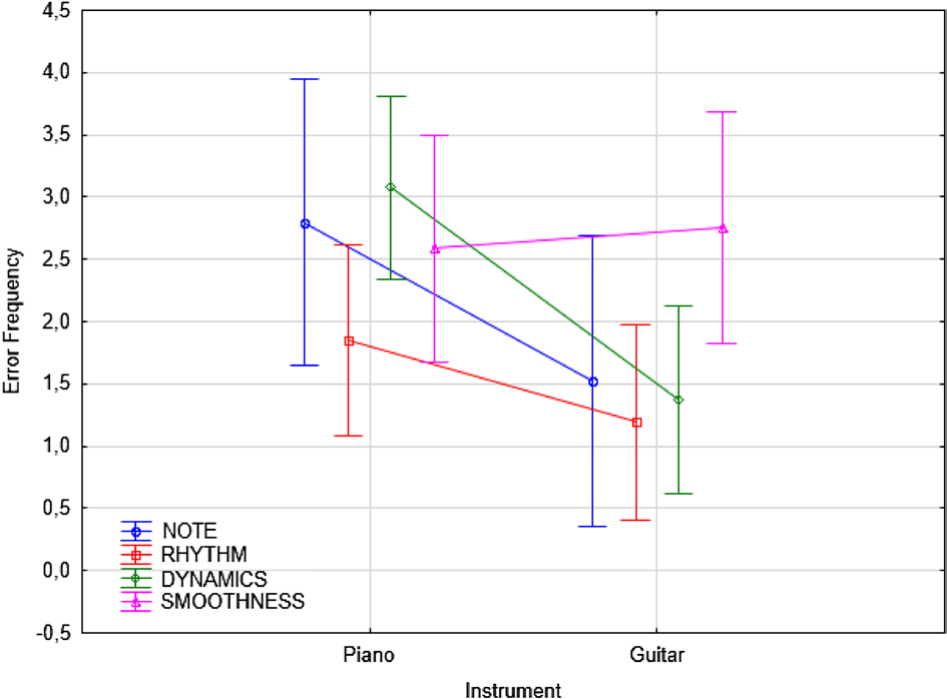
Error frequency observed for the two musical instruments as a function of error type (regardless of the students’ level and learning condition). Error bars indicate standard errors.

The ANOVA also yielded the significance of Instrument x Error type: [F(3, 327 = 7.13; *p* < 0.001]. Post hoc comparisons showed that pianists made significantly (*p* < 0.001) more errors than guitarists, especially of the pitch, rhythm and dynamics types (*p* < 0.001), while there was no difference in the frequency of smoothness errors (*p* = 0.882), as shown in Fig. 4.

**Figure 5:**
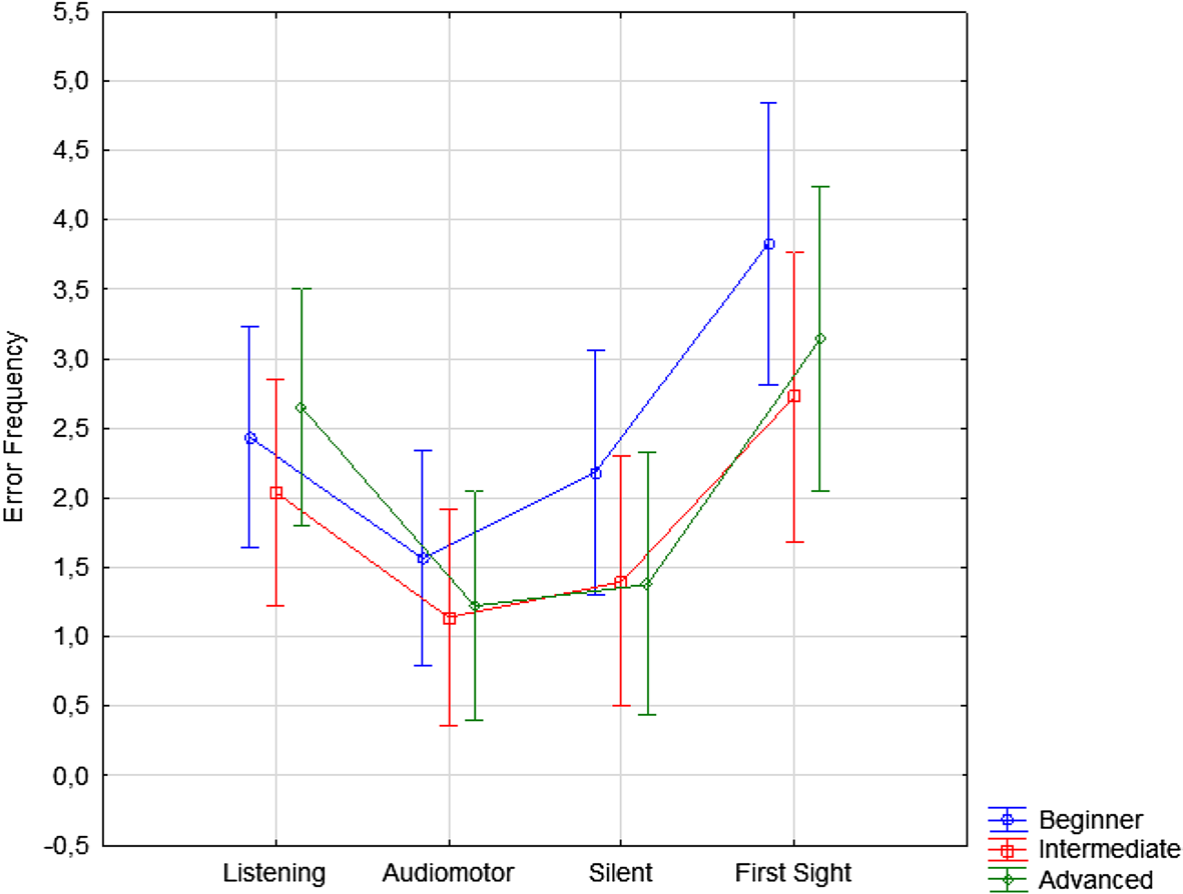
Error frequency observed for the two musical instruments as a function of error type (regardless of the students’ level and learning condition). Error bars indicate standard errors.

The least frequent error type for pianists involved the incorrect reproduction of rhythmic Figs. (1.85, SE = 0.39), which was followed by note (*p* = 0.001), smoothness (*p* < 0.001) and dynamics errors (*p* = 0.011). The most frequent error type for guitarists was lack of smoothness (2.75, SE = 0.47) compared to the other error types (*p* < 0.001). The interaction of instrument x level was significant [F(2, 109) = 4.43; *p* < 0.01]. Among beginners, pianists made more errors (3.34, SE = 0.22) than guitarists (1.67, SE = 0.24), as confirmed by post hoc comparisons (*p* < 0.001). They were followed, in descending order by the number of errors, by advanced pianists (2.29, SE = 0.24) and advanced guitarists (1.91, SE = 0.25), which did not differ significantly (*p* = 0.27), and by intermediate pianists (2.1, SE = 0.24) and intermediate guitarists (1.55, SE = 0.23), which again did not differ statistically (*p* = 0.1). The ANOVA also showed a significant interaction between Condition x Level [F(6,327 = 2.20; *p* = 0.043], illustrated in Fig. 5.

**Figure 6:**
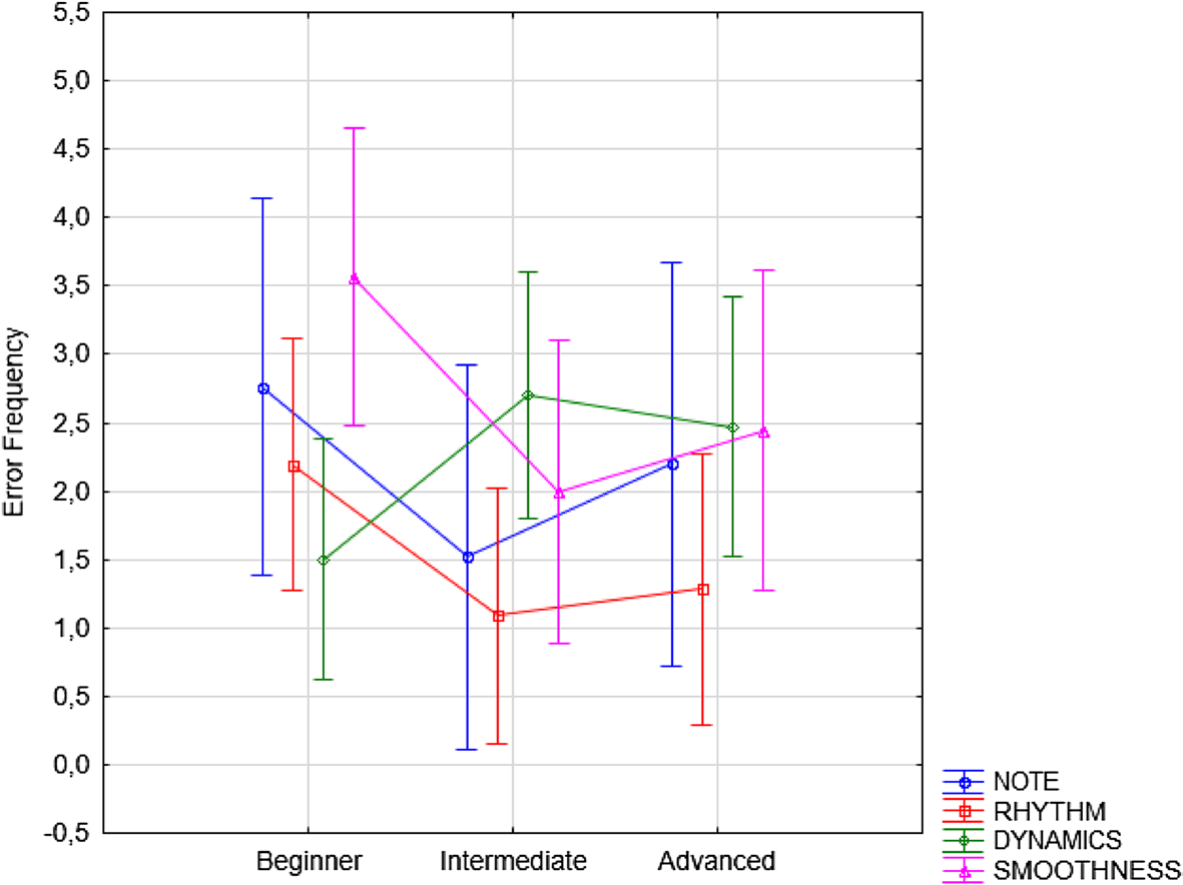
Error frequency observed for the three groups of students as a function of error type (regardless of the instrument played and learning condition). Error bars indicate standard errors.

Post hoc comparisons showed that beginner students (regardless of instrument and error type) committed more errors than more experienced students did in the first-sight (3.83 SE = 0.51), and silent motor conditions (2.19 ES = 0.44), both characterized by a lack of auditory feedback. The further significant interaction between Error type and Level [F(6, 327) = 6,72; *p* < 0.001], and relative post hoc comparisons, showed that beginners (*p* < 0.001) made more errors of all types than advanced students, with the exception of dynamics errors, perhaps because expressiveness cues were very evident in their pieces or because the teachers did not expect a great expressiveness from them yet (see Fig. 6).

**Figure 7:**
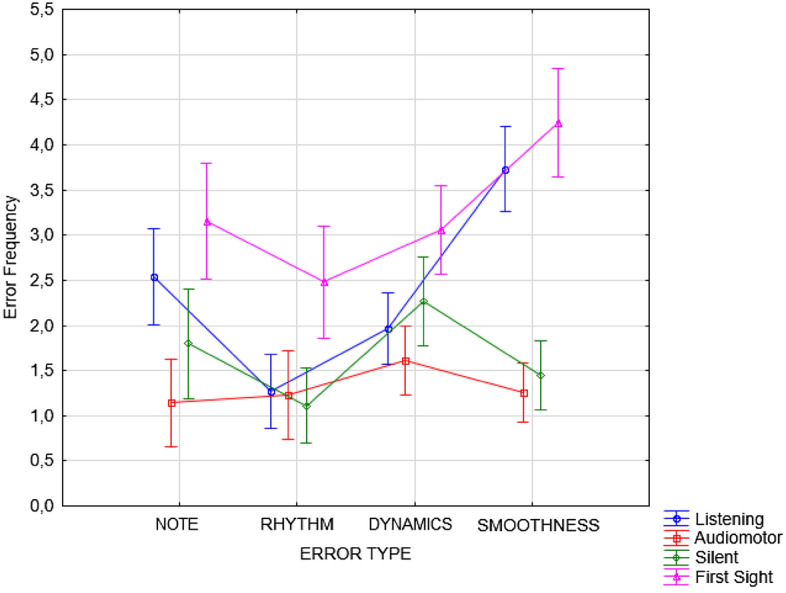
Error frequency observed for the different types of errors committed in the various learning conditions, regardless of the students’ level and instrument played. Error bars indicate standard errors.

The Condition x Error Type interaction proved to be significant [F(9,981) = 7.02; *p* < 0.001]. Post hoc comparisons (*p* < 0.001) showed that the greatest difference in performance was observed between the first-sight and audio-motor conditions; the error rate for all error types was significantly higher in the first of the two conditions (*p* < 0.001). Performance was also significantly better in the silent motor than in the first-sight condition for all error types (*p* < 0.001). The error rate was also lower in the listening than in the first-sight condition for note (*p* = 0.04), rhythm (*p* < 0.001) and dynamics (*p* < 0.001) errors but not for smoothness errors (see Fig. 7). The error rate was higher in the listening condition than in the audio-motor condition only for note (*p* < 0.001) and smoothness (*p* < 0.0001) errors, whereas listening led to effective learning of rhythm and expressiveness (dynamics). The error rate was higher in listening than in silent-motor learning, only for note (*p* < 0.02) and smoothness (*p* < 0.001) errors.

**Figure 8:**
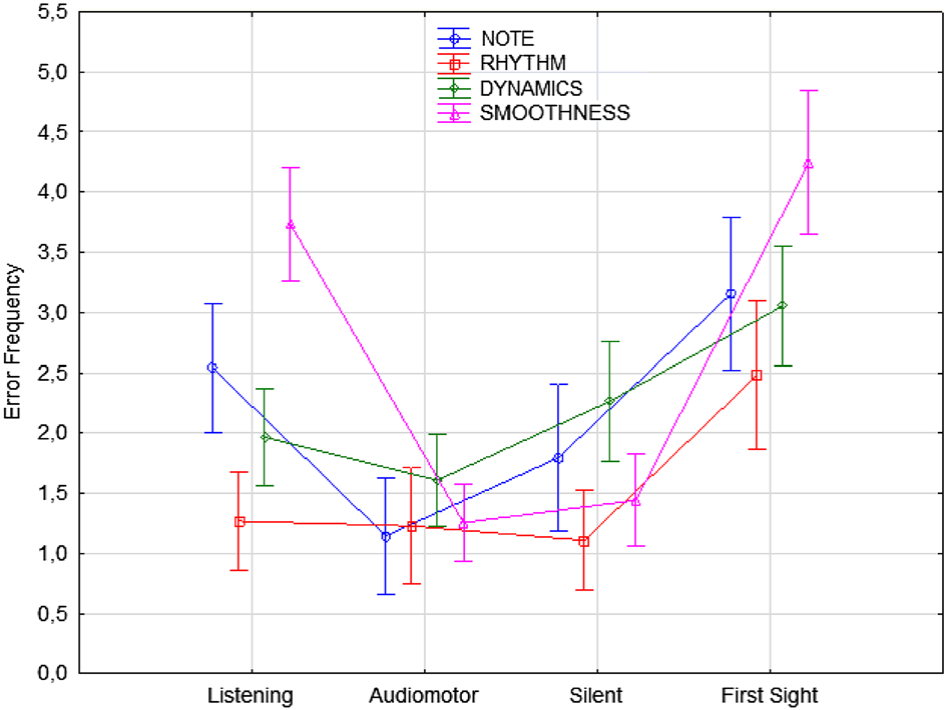
Error frequency observed in the various learning conditions and as a function of error type (regardless of the students’ level and instrument played). Error bars indicate standard errors.

Smoothness errors were committed more frequently in the listening and first-sight conditions than in the two learning conditions involving motor practice (i.e., audio-motor and silent motor conditions, indifferently), as shown in Fig. 8. Smoothness errors were lowest in the audio-motor and silent motor conditions, again with no difference between the two conditions.

Overall, the error rate was higher in the silent motor condition than in the audio-motor condition only for note (and pitch) (*p* < 0.03) and dynamics (expressivity) errors (*p* < 0.04). Errors in dynamics (tempo, expressiveness, articulation, etc.) were higher in the silent motor and first-sight learning conditions than in the listening and audio-motor conditions (both of which provided auditory feedback). Rhythm errors were significantly reduced in each condition compared to the first-sight performance (see Fig. 8).

Other statistical contrasts were not significant. Namely: condition x instrument x level (F = 1.143, *p* = 0.337); condition x error x instrument (F = 0.644, *p* = 0.76); condition x error x level (F = 1.227, *p* = 0.231); condition x error x instrument x level (F = 1.027, *p* = 0.425).

## Discussion

The present study involved a large sample of music students of different proficiency levels to test how ‘auditory feedback’ provided during music playing (A + M +) influenced learning and performance of previously unknown music studies compared to the lack of auditory feedback of a motor ‘silent’ study (A − M +). Furthermore, it was investigated whether prior and repeated listening to piece recordings (A + M −) could improve motor learning in the absence of motor practice on the instrument. The data showed that there was definitely an improvement in performance (compared to reading at first sight, A − M −) after all types of learning, especially after audio-visual learning (A + M +). This proves the validity of the experimental paradigm. Studying or listening to an unfamiliar piece of music (even if only for twelve minutes, once a day, for five days) led to a significantly better performance than not studying at all, which shows that (in real life) if no improvement in performance is observed, it is likely that no studying took place.

Statistical analyses showed that, in this study, pianists made more mistakes than guitarists, regardless of the study condition. This could be due to the reading difficulties caused by the double stave or to a more complicated set of bimanual gestures to be performed by pianists. The most frequent error for pianists concerned dynamics (i.e., the correct observance of tempo, agogics and sound articulation cues), while for guitarists, it concerned smoothness (not jamming). Despite this difference, the results obtained for guitar and piano were quite similar, notwithstanding the major technical and sound production differences between the 2 instruments, such as the presence of piano keys or asymmetrical guitar gestures.

The beginner group committed the highest number of errors, which confirms the key role of experience in musical performance^30^. The most frequently committed error was that of smoothness, i.e., the lack of continuity and regularity in the performance. This category of errors was particularly broad, and the judges reported it every time the student stopped starting again or jammed in the performance, which was a particularly frequent occurrence when there had been no opportunity to study the piece prior to the performance (A − M −). The temporal and spatial precision of actions, such as the fine movements of a pianist's fingers and hand, are necessary for accurate and satisfying performance^31,32^. As argued by Lappe and coworkers^4^ when playing an instrument, these movements are driven by expectations and predictions that are created during years of practice: probably for this reason, beginners were most penalized in performance and especially in reading at first sight. Interestingly, advanced musicians committed significantly more errors of note and smoothness type than intermediate musicians, which might reflect the higher technical difficulty of their studies compared to those assigned to intermediate students. This hypothesis is supported by the evidence that because of the intrinsic structural difficulty of the top-level repertoire (e.g., for number of notes, speed, rhythmic and executive technical complexity), it generally requires a considerably higher amount of hours x day of practice than the simplest repertoire.

### The role of auditory feedback

The data showed that beginners were the only group to be significantly harmed by the absence of acoustic feedback in the home studio. As inexperienced musicians, they based their performance more on control mechanisms via online acoustic feedback. With years of practice, musicians learn to anticipate the auditory consequences of finger movements on the instrument during performance to avoid errors^33^. According to neuroscientists, musicians need to constantly compare these predictions with the actual auditory outcome^34^ and to use auditory feedback to guide error correction and to build the sensorimotor map between movements and their auditory outcomes^13^. The findings that beginners (with on average 3.65 years of musical study) showed the highest error rate (notwithstanding the simplicity of their pieces) indicate the importance of long musical training for the formation of solid audio-motor associations^5,6,26^. Similar to what was previously found with violinists and pianists^23^, three years of academic musical studies seem to be largely insufficient for the development of a feedforward audio-motor system able to predict auditory outcome based on proprioceptive signals. Consistently, the data showed that beginners benefited the most from listening to the piece during performance. Indeed, only beginners were penalized by the lack of auditory feedback in the silent motor (M +) versus audio-motor (A + M +) condition.

Intermediate students made more dynamic errors (including missing or misplaying dynamic, agogic and articulation cues) than other students. This was likely due to the level of pieces. There are numerous dynamic, agogic and articulation cues, but they have not reached a level of performing maturity to be able to return them in their performance.

For advanced music students, no difference in performance was found between the audio-motor (A + M +) and silent conditions (M +), the latter being a condition lacking auditory feedback. This piece of data supports the model proposed by other authors with regard to audio-motor mapping (e.g.^20,21^): once a stable correspondence between musical gestures and produced sounds has been learned, the execution of movements on the instrument would rely more on feedforward control mechanisms. Indeed, experienced musicians are known to have greater auditory imagery skills^35^, which intervene to compensate for the lack of acoustic feedback^36^. As argued by Lashley^37^, acoustic feedback is too slow a means of performance control for regulation of line motor planning of experienced musicians,

Listening previously to the piece (without musical score) for 60 min (over the course of one week) consistently improved the musical performance for both the instrument and student levels. This demonstrates that repeatedly listening to music pieces or melodies can lead to the formation of sound-action associations^8,17,21,38^. Our results fit with the findings reported by Brown & Penhune^22^ on the efficacy of music listening on motor learning.

### Rhythm, expressivity and pitch learning

The 3 types of learning (A − M + , A + M − , A + M +) significantly (and equally) reduced the frequency of rhythm errors with respect to first-sight reading. Indeed, the number of rhythm errors committed in the listening condition (A + M −) or in the silent motor condition (A − M +) was not significantly higher than those committed after audio-motor practice (A + M +). Several studies have shown that because of the strong link between rhythm and movement brain areas^28,39^, musical practice on a mute instrument leads to the learning of the rhythmic structure of a piece. Again, neuroimaging studies^14,17^ have shown that listening to unfamiliar rhythms can activate motor-related areas, such as the motor cortex, basal ganglia and the cerebellum (thus leading to rhythm learning), because of the high interconnection between auditory and motor areas^39^.

In this study, expressivity was significantly transmitted through music listening (A + M −) but was also partly learned through mute motor practice (A − M +). Previous listening to the pieces significantly improved the performance in terms of tempo, agogic and articulation. Dynamics errors were, however, more numerous in the silent-motor condition than in the audio-motor condition; these results are similar to those obtained by Repp^21^, who noted a deterioration in the performance of expressive dynamics in the mute motor condition. However, expressiveness was much better in the silent motor than in the first sight condition, thus suggesting that musicians were capable of generating expressiveness of their performance in the absence of auditory feedback during learning due to an internal audio-motor and kinesthetic representation, as also shown by Repp^21^.

In addition, fewer note/pitch errors were overall committed in the listening condition (A +) than in the first-sight condition (A − M −). These data are strongly coherent with the findings reported by Brown and Penhune^22^, according to which sensory information from listening is transformed into motor production in musicians. It also confirms the presence of sound-action translation mechanisms in the musician’s mind, in which brain circuits similar to those activated in actual movement are activated by imagining the movements^14,40^. The number of note errors was, however, lower in the audio-motor (A + M +) than in the silent-motor condition (A − M +), and especially the listening (A + M −) conditions. This finding suggests that during audiomotor practice, sound gesture associations are repeatedly reactivated, leading to improved intonation and to the formation of predictive feedforward mechanisms based on audio visuomotor connections^23^.

## Conclusions

Overall, these results indicate that musical performance is multifaceted and based on skills and processes in close interaction but partly independent; for example, the rhythmic component was not affected by the lack of acoustic feedback, unlike pitches and dynamics. Acoustic feedback has proven to be particularly important for the musical learning of inexperienced students. Listening attentively and repeatedly to a recorded execution led to a significant improvement in performance for beginners and advanced students, indicating how closely sounds are associated with motor gestures in the experienced brain. The results of this study highlight the importance of listening in music learning: this aspect is sometimes unjustly neglected in teaching, which is more focused on the development of agility and motor dexterity.

## Study limits

One possible limitation of the study is that it only involved piano and guitar instruments and no, for example, strings or winds. It would be interesting in the future to observe the possible presence of instrument-related differences in the number and quality of errors as a function of different learning variables. The other possible limitation is the lack of an objective control of the students’ practice during the weeks. This limitation is compensated for by the notion that the students were recruited on a voluntary basis within an educational/institutional setting, were carefully trained to monitor with a timer the time of study and were monitored by their conservatory teachers and the youngest by their parents at home.

## Supplementary Information

Supplementary Information.

## Data Availability

The authors confirm that the data supporting the findings of this study are available within the article. Other information is available on request from the corresponding author.
