# Supplementary material for: The role of auditory feedback in the motor learning of music in experienced and novice performers
Source: Sci Rep. 2022 Nov 17;12:19822. doi: 10.1038/s41598-022-24262-x (PMC9671877; doi:10.1038/s41598-022-24262-x)

Appendix 1 of paper "The role of auditory feedback in the motor learning of music in experienced and novice performers"

By Maria Luciani, Alessandra Cortelazzo & Alice Mado Proverbio

This supplementary material contains some examples of pieces assigned to students of different proficiency level, for the two musical instruments.

BEGINNER PIECE FOR PIANO

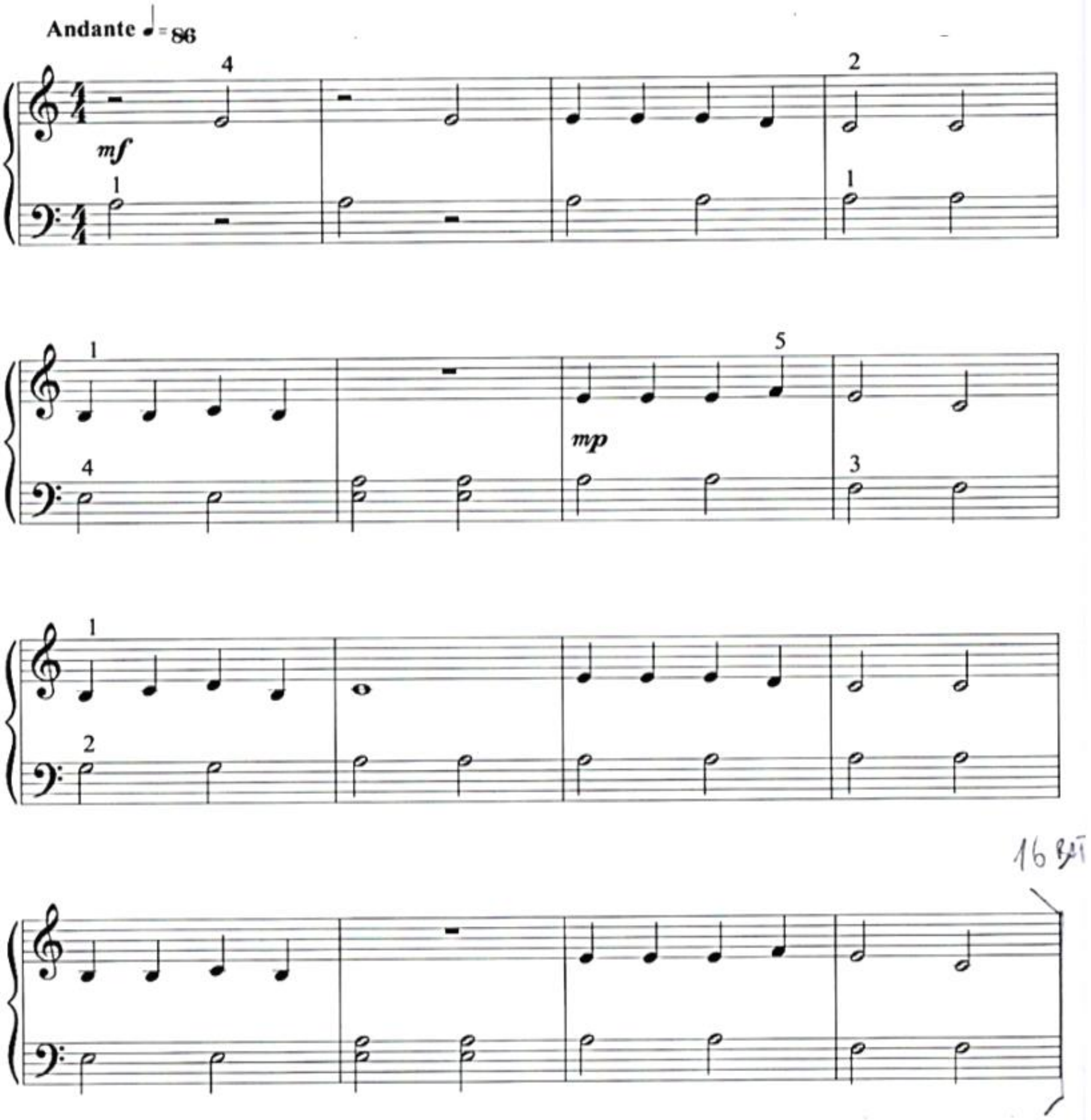

BEGINNER PIECE FOR GUITAR

3 FURIANT

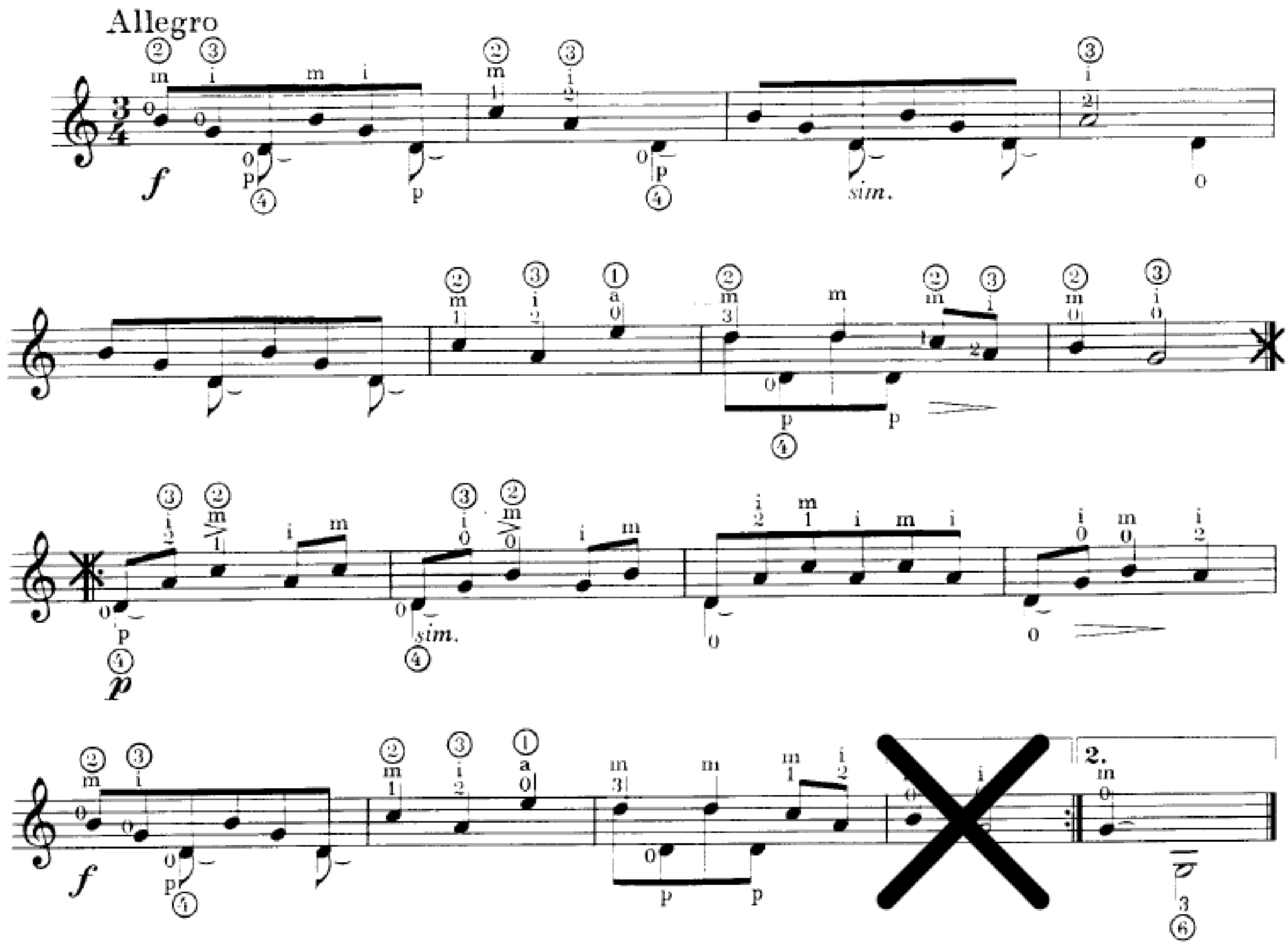

INTERMEDIATE PIECE FOR PIANO

Matin froid

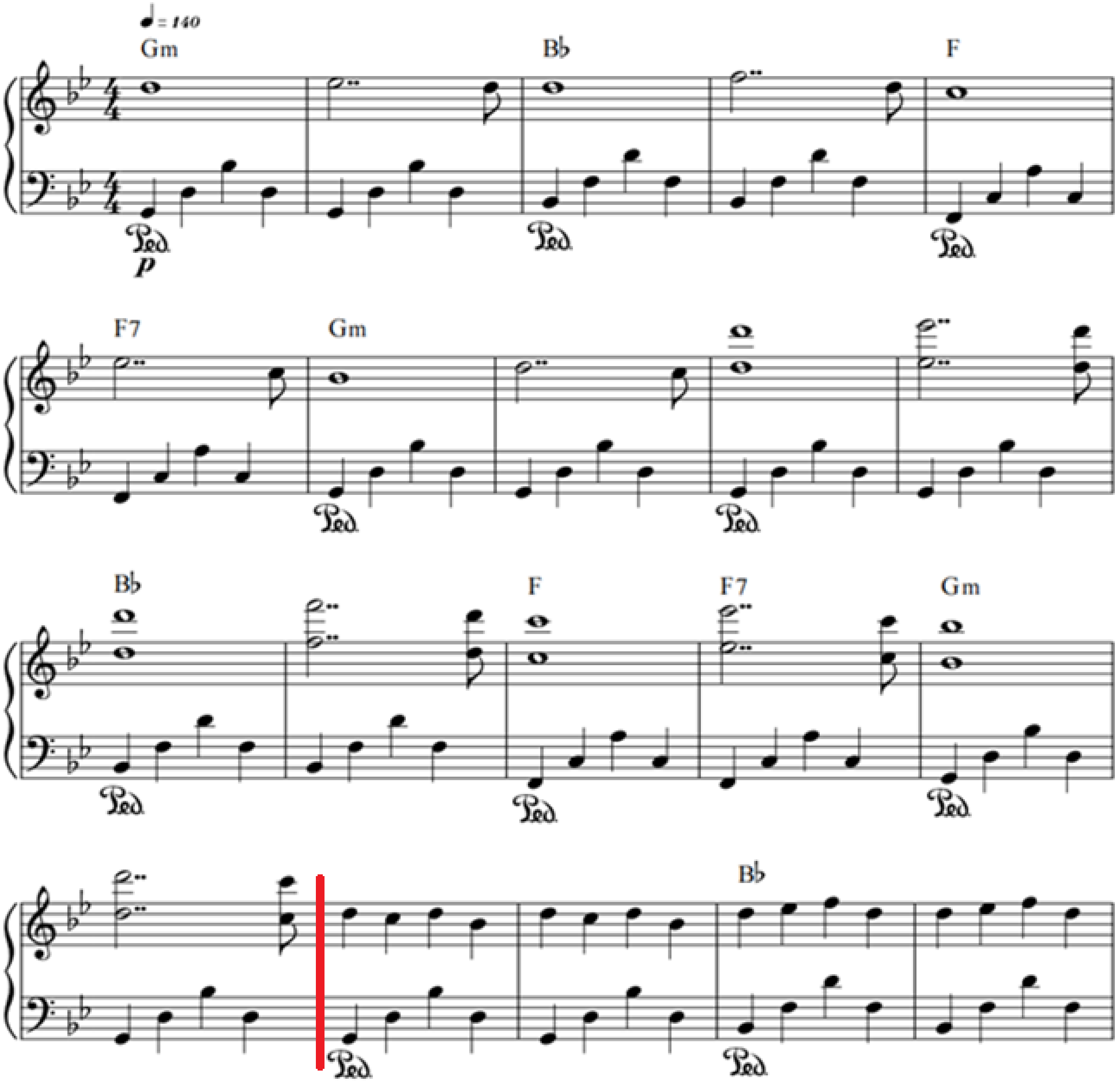

INTERMEDIATE PIECE FOR GUITAR

ALLEGRETTO

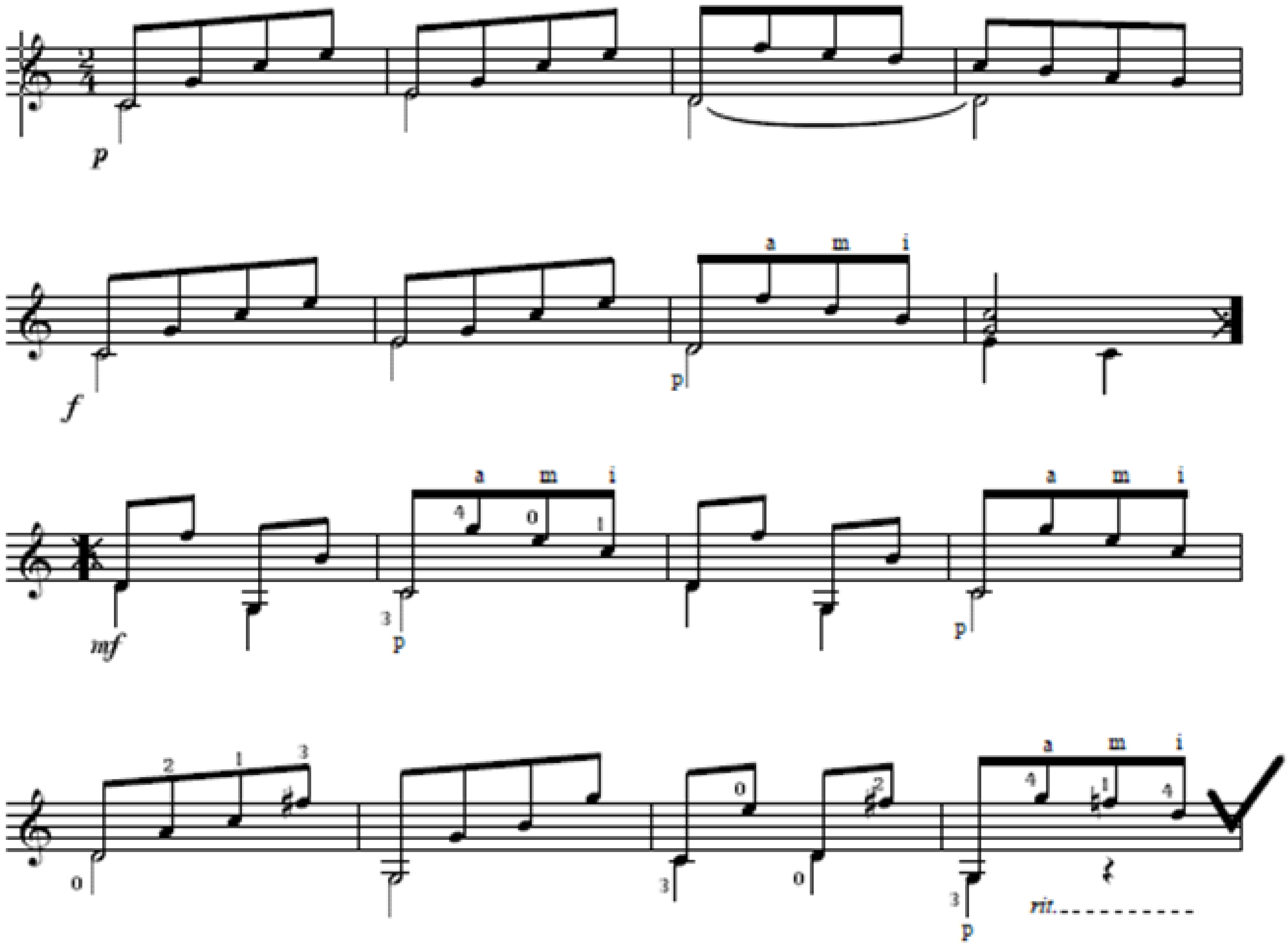

ADVANCED PIECE FOR PIANO

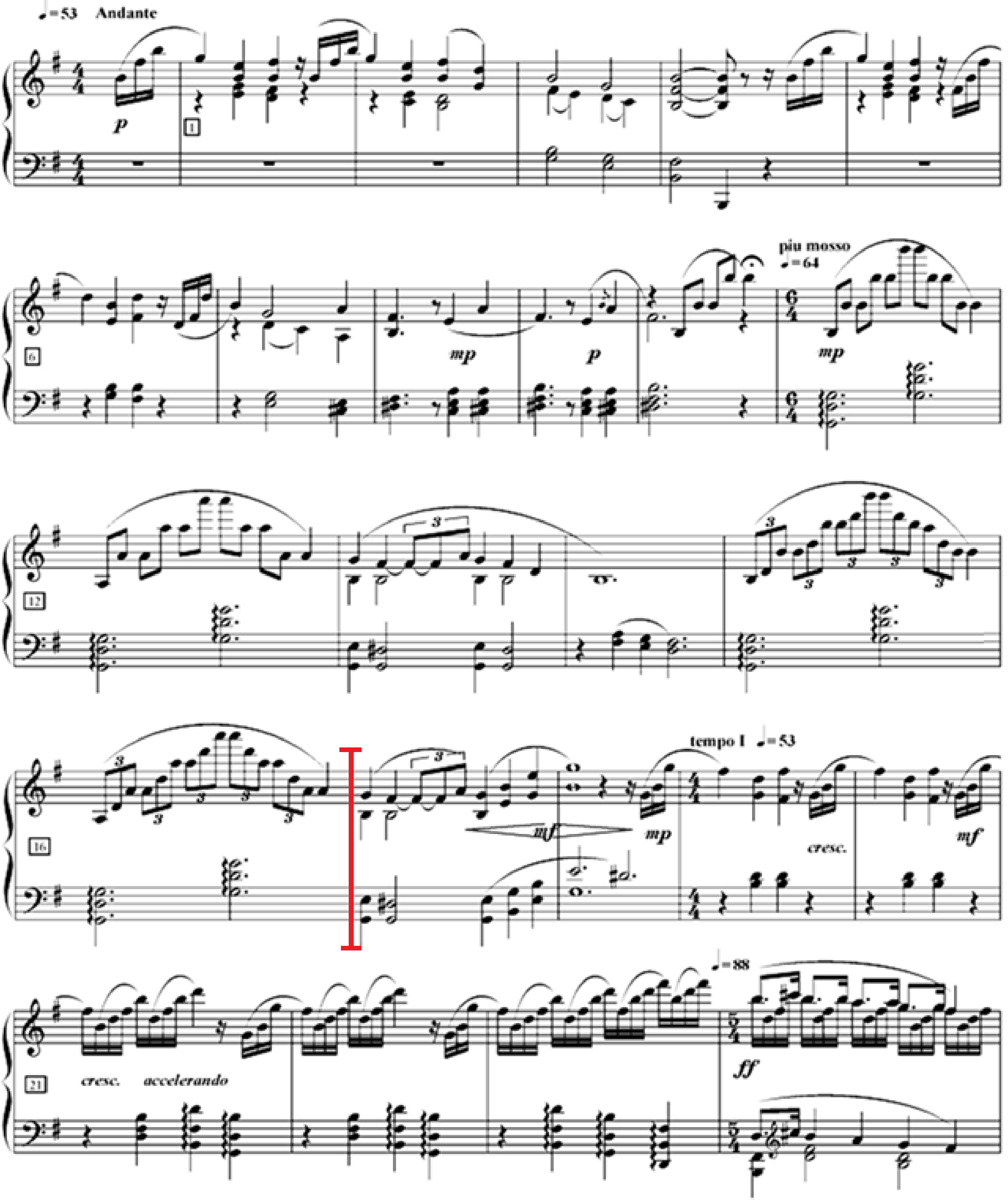

ADVANCED PIECE FOR GUITAR

Etude No 2

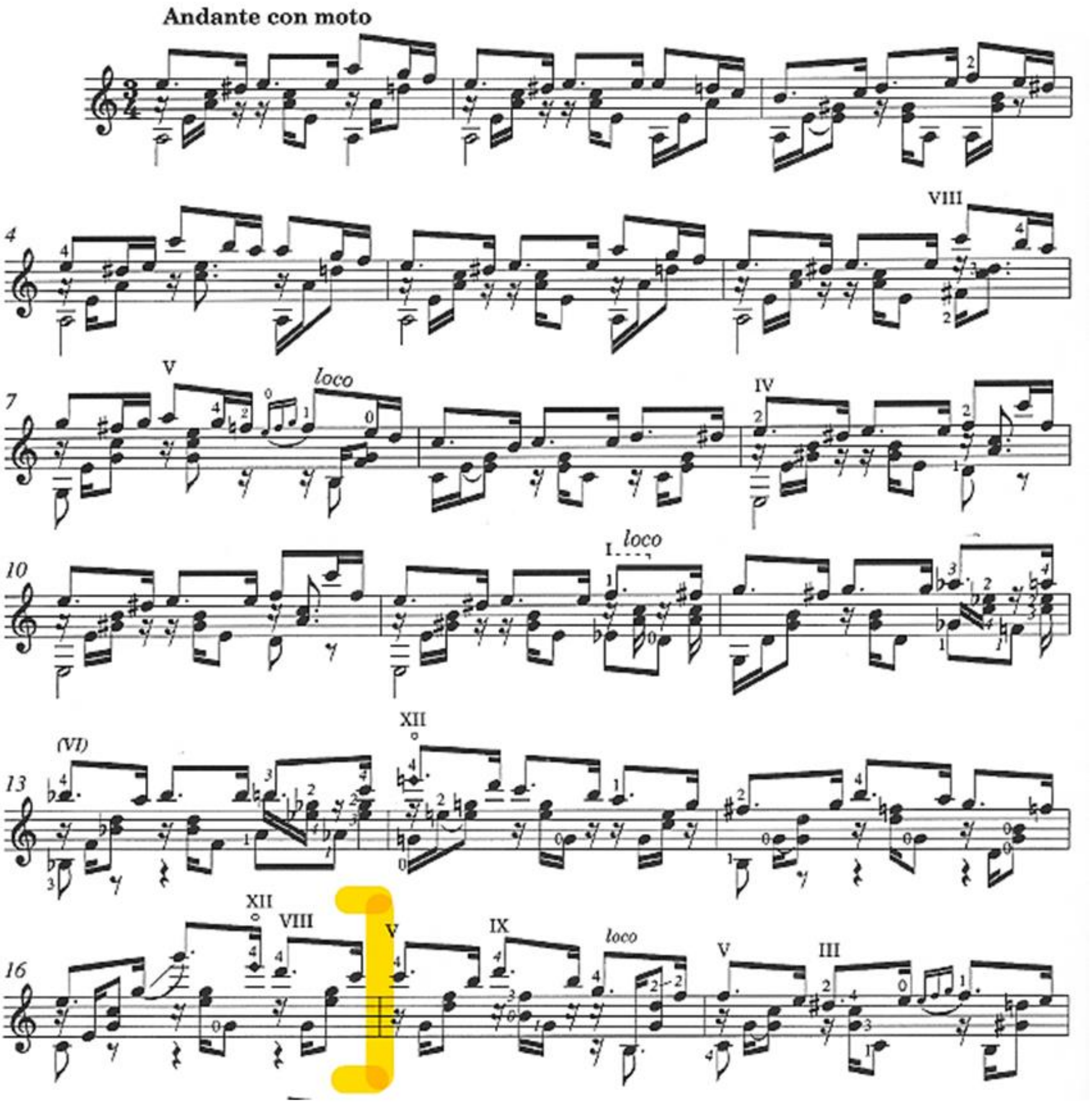

Supplement: Supplementary file 1 — Supplementary Information. [file 41598_2022_24262_MOESM1_ESM.pdf]
